# Missed opportunity: low uptake of VMMC among men attending the OPD of a public health facility offering free VMMC services in Uganda

**DOI:** 10.1186/s12889-023-15056-5

**Published:** 2023-01-18

**Authors:** Ruth Nyaiti Kiyai, David Livingstone Ejalu, Derrick Kimuli

**Affiliations:** 1 Faculty of Health Sciences, Uganda Martyrs University, P.O. Box 5498, Kampala, Uganda; 2 Galen Holdings Group, P.O.Box, 7714, Kampala, Uganda; 3 Faculty of Science and Technology, Department of Health Sciences, Cavendish University Uganda, P.O Box 33145, Kampala, Uganda

**Keywords:** Circumcision, Wakiso Uganda, VMMC, Outpatient departments

## Abstract

**Background:**

Studies in various countries including Uganda and Kenya have shown a much lower incidence of the human immunodeficiency virus (HIV) among men that underwent voluntary medical male circumcision (VMMC) compared to uncircumcised men. Wakiso district, the district with the highest prevalence of HIV in Uganda (7%), has a very low estimated proportion of men who have undergone VMMC (30.5%). Within the district, various public health facilities provide free VMMC services. This study examined the prevalence and factors associated with the uptake of VMMC among men attending the outpatient department (OPD) of a public facility offering VMMC services.

**Methods:**

We conducted a cross-sectional study between July to August 2021 using a sample of men attending the OPD at Kira Health Centre IV. We defined VMMC uptake as the removal of all or part of the foreskin of the penis by a trained healthcare professional. We determined factors independently associated with VMMC uptake using a modified Poisson regression analysis with robust standard errors at a 5% statistical significance level. Adjusted prevalence risk ratios (APRR) were reported as the measure of outcome.

**Results:**

Overall, 389 participants were enrolled in the study. The mean age of the participants was 27.2 (standard deviation ± 9.02) years. The prevalence of VMMC uptake was 31.4% (95% Confidence Interval [CI] 26.8–36.2). In the adjusted analysis, the uptake of VMMC among men attending the OPD of Kira HC IV was less likely among married participants compared to unmarried participants (APRR 0.64, 95% CI 0.48–0.88), among participants from Western tribes (APRR 0.50, 95% CI 0.41–0.86) or Eastern tribes (APPR 0.31, 95% CI 0.13–0.72) compared to participants from the Central tribes and among participants who didn’t disclose their sexual partner number compared to those that had one or no sexual partner (APRR 0.62, 95% CI 0.40–0.97). On the other hand, the prevalence of uptake of VMMC was 7 times among participants who were aware of VMMC compared to those who were not aware of VMMC (APRR 7.85 95% CI 1.07–9.80) and 2.7 times among participants who knew their HIV status compared to those that didn’t know (APRR 2.75, 95% CI 1.85–4.0). Also, the uptake of VMMC was 85% more among participants who knew that Kira HC IV provided free VMMC services compared to those that didn’t (APRR 1.85, 95% CI 1.85–4.08).

**Conclusion:**

VMMC among men attending the OPD at the largest public healthcare facility proving free VMMC services in Kira Municipality was low. The OPD may provide a quick win for improving VMMC uptake. Collaborative efforts among the administration of Kira HC IV, the Ministry of Health and VMMC implementation partners could work towards developing health-facility-based strategies that can improve VMMC awareness and uptake with emphasis on the OPD.

## Background

Voluntary Medical Male Circumcision (VMMC) is the removal of all or part of the foreskin of the penis by a trained healthcare professional. Although circumcision (i.e. the removal of all or part of the foreskin of the penis) is a long-standing practice, it has been majorly performed for religious or cultural and not medically [1, 2]. This often poses a higher risk for complications, the spread of diseases and adverse events. VMMC was adopted as part of the human immunodeficiency virus (HIV) prevention strategies due to its efficacious and cost-effectiveness in reducing heterosexual transmission of HIV [3, 4]. In such settings as Uganda [5] and Kenya [6], study findings showed a much lower incidence of HIV among those males that underwent VMMC compared to those that did not. Also, in Uganda, Kenya and the United Republic of Tanzania, which are part of 14 VMMC priority countries [7], of all circumcisions conducted, VMMC contributes to as little as 10%, 26% and 37% respectively in some regions [8]. Consequently, the implementation of VMMC programs is inhibited by existing cultural and religious practices upon which strategies need to be developed to improve VMMC. The majority of religious and cultural circumcisers are not medically trained on infection control procedures, eligibility, management of adverse events, wound care and quality control procedures for circumcision [1, 8].

The latest (2016) demographic and health survey (DHS) data show that, overall, less than half of men in Uganda (46%) are circumcised, 22% of which were circumcised through VMMC [9]. VMMC in Uganda is one of the primary strategies adopted for the reduction of new HIV infections [10, 11]. As such, the majority of the public higher-level health centres in the country provide free VMMC services. However, circumcision in the country largely varies due to the differences in religious and ethnic values. Consequently, circumcision is higher among Muslims and circumcision-ethnic tribes such as those from the Eastern region of the country compared to the non-Muslims and tribes in the central, northern and east-central regions of the country [9].

Wakiso district, the district with the highest prevalence of HIV in Uganda (7%) [12] has a very low estimated proportion of men who have undergone VMMC (30.5%) [9]. VMMC is estimated to reduce the risk of HIV transmission by up to 60% [7]. Therefore, the low coverage of VMMC in the district is a likely contributor to the prevalence of HIV and a possible increase in HIV prevalence in the future. The MoH has made massive investments in social behavioural change communication strategies [10] and also provides free VMMC services at many public health facilities [11]. The outpatient departments (OPD) at such facilities can be leveraged to boost VMMC rates. However, the proportion of eligible men attending the OPD who have undertaken VMMC is unknown. Moreover, the factors influencing VMMC uptake among men attending the OPD of a public health facility that provides free VMMC have not been comprehensively studied. This study examined the prevalence and factors associated with VMMC uptake in an OPD at one of the largest public health facilities offering free VMMC services in Wakiso district.

## Methods

### Study design and setting

The study was an analytical cross-sectional study because it enabled the collection of data at a single point in time. The design was suitable because, due to the limited amount of time and resources, the design ensures a faster turn-around time at an affordable cost. This design also allowed for the ability to examine both the dependent and independent variables at the same time. The study was conducted from July to August 2021 at Kira Health Centre IV in Kira Municipality, Wakiso district, in Uganda. Kira Municipality is bordered by Lake Victoria to the south, Gayaza to the north, Kampala to the west, Mukono to the east, and Kasangati to the north-west.

Kira Health Centre IV is the second largest public health facility in Wakiso district. The facility has a catchment population of about 470,000 people and has only just recently been upgraded to Health Centre IV status [13]. This is due to the demand by the increase in the population of the area making it the busiest health facility in Kira Municipality, the second busiest municipality in Uganda. The health centre is located 14 kms from Kampala at global position satellite coordinates Latitude 0.45336, Longitude 32.63405. The facility has the highest catchment population in Kira Municipality and it offers free VMMC services. This implies that the facility has a great capacity and potential to extend VMMC services to the majority of the men seeking health services.

### Study population

The study population was all adult men attending the OPD clinic at Kira Health Centre IV. This helped to determine the proportion of circumcision of men who are attending free health services at a public health facility that offers “free” VMMC services. Men 15–49 years are a targeted priority age group for circumcision due to their higher risk of acquiring HIV and other sexually transmitted infections [11].

### Eligibility criteria

#### Inclusion criteria

All men aged 15–49 years attending OPD at Kira Health Centre IV who were residents of Kira Municipality that provided informed consent were invited to participate in the study because this age is the target population for VMMC. For participants who were aged less than 18 years, informed consent was sought from their parents or caretakers.

#### Exclusion criteria

All men who were severely sick or had a physical impairment affecting speech or sight were not involved in the study because of perceived challenges in the ability to consent or respond to the questionnaire.

### Research variables

The outcome variable for the study was the uptake of VMMC. An individual was considered to have took up VMMC if by the time of the study: (a) he had all or part of his foreskin of their penis removed and (b) if the removal was by a trained healthcare professional in a healthcare setting. The independent variables for the study were predisposing factors (age, marital status, education, income, tribe, employment, physical activity status), Enabling factors (knowledge of VMMC, its cost, and advantages, perceived risk of contracting HIV and impact of sexual life, anticipated pain from VMMC and wound healing) and Need factors (knowledge of HIV status, number of sexual partners, external influence and use of other HIV prevention methods).

#### Sample estimation

The sample size for the study was 389 calculated using Kish’s formula for estimating the sample size for a single proportion [14]. The sample calculation considered precision of 5%, a normal standard deviation of 1.96 corresponding to a 95% confidence interval (CI), an estimated proportion of VMMC of 40% [15] and a non-response rate of 5%.

#### Sampling procedure

The study employed a systematic random sampling method to select participants. This method was used because it is appropriate for preventing selection biases and ensuring the generalizability of the findings. The sampling was executed using the following procedure. First, the average OPD attendance of men aged 15 – 49 years at Kira Health Centre IV was established using the records at the facility and divided by the desired daily sample size to establish a sampling interval (19). Simple random sampling was then used to select a random starting number within the first interval and thereafter the sampling interval was used to select subsequent eligible participants until the desired daily sample was reached. Where an eligible participant did not provide informed consent, the next eligible participant would be chosen.

### Data management and analysis

The study involved the collection of quantitative data directly from the participants through confidential interviews to obtain first-hand information directly from the participants. A pre-tested questionnaire designed to answer the objectives of the study was used. After the pretesting and finalization of the questionnaire, it was electronically designed using the free online electronic application Open Data Kit (ODK™) [16]. The data collected was exported into Microsoft Excel and analysed using STATA 15 [17].

In univariate analysis, demographic characteristics data were analysed descriptively using frequencies and percentages for categorical data. For numeric data such as age that was normally distributed, means and standard deviation were used to summarize findings. For bivariate analysis, a chi-squared test (*x*^2^) was used to assess statistically significant differences between the uptake of VMMC and categorical variables for expected cell counts of five and beyond otherwise, Fisher’s test was used. The uptake of VMMC was categorized as a binary variable Yes (1) or No (0). Regression was used to compute unadjusted prevalence risk ratios (UPRR) at 5% statistical significance. A forward multivariable analysis using a modified Poisson regression analysis robust standard error variance was used to compute adjusted prevalence risk ratios (APRR) with corresponding 95% Confidence Intervals (CI). Variables with a probability value (*P*-value) of less than 5% were considered statistically significant. The model parsimony was assessed using the Akaike Information Criteria (AIC).

## Results

### *Characteristics of study participants*

Overall, 404 persons were approached of whom 389 participants agreed to be enrolled in the study, giving a study a response rate of 96.3%. The mean age of the participants was 27.2 (standard deviation ± 9.0) years. The age range of the participants was 15—49 years. Of the participants, 55.5% were less than 27 years, 57.8% were unmarried and 36.5% were of tribes from the central region of Uganda. Of the participants 43.2% had completed secondary education, 48.1% had an income of less than 150,000 Ugx, 47.8% were employed informally and 36.8% were of the Catholic religion. Table 1 presents the distribution of participant characteristics.

**Table 1:** Characteristics of the study participants

Variable	Category	Frequency *n* = 389	Per cent (% = 100)
Age	≤ 27 years	216	55.5
	> 27 years	173	44.5
Marital status	Unmarried	225	57.8
	Married	164	42.2
Tribe origin	Central	142	36.5
	Western	84	21.6
	Northern	50	12.9
	Eastern	52	13.4
	Southern	61	15.7
Highest level of education	None	26	6.7
	Primary	97	24.9
	Secondary	168	43.2
	University/Tertiary	98	25.2
Income	< 150, 000 Ugx	187	48.1
	150,000 – 500,000 Ugx	135	34.7
	> 500,000 Ugx	67	17.2
Employment type	Unemployed	130	33.4
	Formal	73	18.8
	Informal	186	47.8
Religion	Born again	83	21.3
	Catholic	143	36.8
	Muslim	71	18.3
	Protestant	75	19.3
	Other Religions	17	4.4

#### Predisposing factors associated with VMMC uptake

The study findings showed that overall, only 31.4% (95% CI 26.8 – 36.2) of the participants had undergone VMMC. Of the participants who had undergone VMMC 59.8% were aged ≤ 27 years, 66.4% were unmarried, 43.4% were from tribes in the central region, 43.4% had completed secondary education, 51.6% had a monthly income of less than 150, 000 Ugx, 38.5% were employed formally or self-employed, and 41.8% were of the Catholic religion. In bivariate analysis, marital status (*p* = 0.021), tribe origin (*p* < 0.001), education (*p* = 0.003) and employment (*p* = 0.004) were the only predisposing factors found to be statistically significantly associated with the uptake of VMMC. Table 2 presents the relationship between predisposing factors and VMMC uptake.

**Table 2:** Bivariate analysis of predisposing factors and VMMC uptake

Demographics	Category	Overall (*n* = 389) ¥	Participant underwent VMMC		*P*-Value
			No (*n* = 267) **¥**	Yes (*n* = 122) **¥**	
Age	< = 27	216 (55.5)	143 (53.6)	73 (59.8)	0.248
	> 27	173 (44.5)	124 (46.4)	49 (40.2)	
Marital status	Unmarried	225 (57.8)	144 (53.9)	81 (66.4)	0.021*
	Married	164 (42.2)	123 (46.1)	41 (33.6)	
Tribe origin	Central	142 (36.5)	89 (33.3)	53 (43.4)	< 0.001***
	Western	84 (21.6)	65 (24.3)	19 (15.6)	
	Northern	50 (12.9)	32 (12.0)	18 (14.8)	
	Eastern	52 (13.4)	47 (17.6)	5 (4.1)	
	Southern	61 (15.7)	34 (12.7)	27 (22.1)	
Highest level of education	None	26 (6.7)	23 (8.6)	3 (2.5)	0.005**
	Primary	97 (24.9)	73 (27.3)	24 (19.7)	
	Secondary	168 (43.2)	115 (40.1)	53 (43.4)	
	University/Tertiary	98 (25.2)	56 (21.0)	42 (34.4)	
Monthly Income	< 150, 000 Ugx	187 (48.1)	124 (46.4)	63 (51.6)	0.230
	150,000– 500,000 Ugx	135 (34.7)	100 (37.6)	35 (28.7)	
	> 500,000 Ugx	67 (17.2)	43 (16.1)	24 (19.7)	
Employment type	Formal	130 (33.4)	83 (31.1)	47 (38.5)	0.044*
	Informal	73 (18.8)	45 (16.9)	28 (23.0)	
	Self-employed	186 (47.8)	139 (52.1)	47 (38.5)	
Religion	Born again	83 (21.3)	45 (16.9)	38 (31.2)	0.053
	Catholic	143 (36.8)	92 (34.5)	51 (41.8)	
	Muslim	71 18.3)	67 (25.1)	4 (3.3)	
	Protestant	75 (19.3)	53 (19.9)	22 (18.0)	
	Other Religions	17 (4.4)	10 (3.8)	7 (5.7)	

¥ Column percentages shown in brackets Significant results at

***0.1%

**1% and

*5%

#### Enabling factors associated with VMMC uptake

Of the participants who had undergone VMMC, 56.6% were frequently active, 99.2% were aware of VMMC, 75.4% were aware that VMMC was free at Kira HC IV, 82.0% knew their HIV status, 63.1% could not be influenced by other parties to uptake VMMC and 59.0% thought that the COVID-19 situation inhibited their ability to seek healthcare. In bivariate analysis, VMMC awareness (*P* < 0.001), knowledge of free VMMC at Kira HC IV (*p* < 0.001), knowledge of HIV status (*p* < 0.000), other influences on VMMC decision making (*p* = 0.001) and the perception that the COVID-19 situation inhibited access to health services (*p* = 0.036) were the only enabling factors that were statistically significantly associated with the uptake of VMMC. Physical activity was the only enabling factor that was not associated with VMMC uptake. Table 3 presents the relationship between enabling factors and VMMC uptake.

**Table 3:** Bivariate analysis of enabling factors and VMMC uptake

Enabling factor	Category	Overall (*n* = 389) ¥	Participant underwent VMMC		*P*-Value
			No (*n* = 267) **¥**	Yes (*n* = 122) **¥**	
Participant is frequently physically active	No	184 (47.3)	131 (49.1)	53 (43.4)	0.303
	Yes	205 (52.7)	136 (51.0)	69 (56.6)	
Participant was aware of VMMC	No	48 (12.3)	47 (17.6)	1 (0.8)	< 0.001***
	Yes	341 (87.7)	220 (82.4)	121 (99.2)	
Participant was aware of free VMMC services at Kira HC IV	No	181 (46.5)	151 (56.6)	30 (24.6)	< 0.001***
	Yes	208 (53.5)	116 (43.5)	92 (75.4)	
Participant knows their HIV status	No	179 (46.0)	157 (58.8)	22 (18.0)	< 0.001***
	Yes	210 (54.0)	110 (41.2)	100 (82.0)	
Participant can be/was influenced by a relative to undergo VMMC	No	287 (73.8)	210 (78.7)	77 (63.1)	0.001**
	Yes	102 (26.2)	57 (21.4)	45 (36.9)	
Participant thinks that the COVID-19 situation at the health facility affected their health-seeking pattern	No	190 (48.8)	140 (52.4)	50 (41.0)	0.036*
	Yes	199 (51.2)	127 (47.6)	72 (59.0)	

¥Column percentages shown in brackets significant results at

***0.1%

**1% and

*5%

#### Need factors associated with VMMC uptake

Of the participants who had undergone VMMC, 73.0% had one or no sexual partner, 82.0% didn’t anticipate any future risky sexual behaviour, 70.0% had a low self-perceived risk of contracting HIV and 58.2% used other HIV prevention methods. In the bivariate analysis, the only need factor that was statistically significantly associated with the uptake of VMMC was the participant’s number of sexual partners (*p* < 0.001). Table 4 presents the relationship between need factors and VMMC uptake.

**Table 4:** Bivariate analysis of need factors and VMMC uptake

Variable	Category	Overall (*n* = 389) ¥	Participant underwent VMMC		*P*-Value
			No (*n* = 267) **¥**	Yes (*n* = 122) **¥**	
Number of active sexual partners	One or none	226 (58.1)	137 (51.3)	89 (72.9)	< 0.001***
	More than one	96 (24.7)	75 (28.1)	21 (17.2)	
	Not disclosed	67 (17.2)	55 (20.6)	12 (9.8)	
Participant anticipates a future risky sexual behaviour	No	314 (80.7)	214 (80.2)	100 (82.0)	0.893
	Not sure	39 (10.0)	28 (10.5)	11 (9.0)	
	Yes	36 (9.3)	25 (9.4)	11 (9.0)	
Participant self-assessed risk of contracting HIV	High	24 (6.3)	17 (6.4)	7 (5.7)	0.620
	Low	281 (72.2)	196 (73.4)	85 (69.7)	
	None	84 (21.6)	54 (20.2)	30 (24.6)	
Participant uses other HIV prevention methods	No	137 (35.2)	97 (36.3)	40 (32.8)	0.345
	Some times	45 (11.6)	34 (12.7)	11 (9.0)	
	Yes	207 (53.2)	136 (50.9)	71 (58.2)	

¥Column percentages shown in brackets significant results at

***0.1%

#### Multivariable analysis of the factors associated with VMMC uptake

Table 5 below shows the results from the unadjusted and adjusted analysis. Findings from the multivariable analysis show that respondents who were married were 36% less likely to uptake VMMC services compared to those who were not married (APRR 0.64, 95% CI 0.48–0.88, *p* = 0.006). Uptake of VMMC services was generally higher among respondents who came from the central region compared to other regions. Findings in this regard show that participants from Western tribes were 50% less likely to use the VMMC services, (APRR 0.50, 95% CI 0.41—0.86, *p* = 0.006); participants from the East were 69% less likely (APPR 0.31, 95% CI 0.13 – 0.72, *p* < 0.007) compared to participants from the Central tribes.

**Table 5:** Multivariable analysis of the study participant characteristics and VMMC uptake

Variable	Category	Unadjusted Prevalence Risk Ratio (UPRR)	95% Confidence Interval	*P*—Value	Adjusted Prevalence Risk Ratio (APRR)	95% Confidence Interval	*P*—Value
Marital status	Unmarried	1			1		
	Married	0.69	0.51—0.95	0.024	0.64	0.47—0.88	0.006**
Tribe origin	Central	1			1		
	Western	0.57	0.37—0.88	0.012	0.50	0.41—0.86	0.006**
	Northern	0.91	0.61—1.37	0.664	0.91	0.63—1.29	0.580
	Eastern	0.240	0.10—0.57	0.001	0.31	0.13—0.72	0.007**
	Southern	1.33	0.73–2.46	0.354	1.24	0.63 – 2.12	0.345
Employment type	Formal	1			1		
	Informal	1.10	0.61–1.99	0.755	0.94	0.64—1.38	0.765
	Self-employed	0.6	0.37–0.98	0.038	0.83	0.60—1.14	0.243
Highest level of education	None	1			1		
	Primary	2.14	0.70–6.58	0.182	1.61	0.51–5.05	0.415
	Secondary	2.73	0.92 – 8.12	0.070	1.63	0.53—5.02	0.394
	University/Tertiary	3.71	1.25—11.05	0.018	1.72	0.54—5.45	0.353
Participant was aware of VMMC	No	1			1		
	Yes	17.03	2.43—19.38	0.004	7.85	1.07—9.80	0.043*
Participant was aware of free VMMC services at Kira HC IV	No	1			1		
	Yes	2.67	1.86—3.83	< 0.001	1.81	1.31- 2.50	< 0.001***
Participant knows their HIV status	No	1			1		
	Yes	3.87	2.55—5.89	< 0.001	2.75	1.85—4.08	< 0.001***
Participant can be/was influenced by a relative to undergo VMMC	No	1			1		
	Yes	1.64	1.23—2.20	0.001	1.19	0.94—1.51	0.155
Participant thinks that the COVID-19 situation at the health facility affected their health-seeking pattern	No	1			1		
	Yes	1.13	0.82—1.55	0.448	0.99	0.77–1.29	0.964
Number of active sexual partners	One or none	1			1		
	More than one	0.56	0.37—0.84	0.005	0.75	0.52—1.08	0.125
	Not disclosed	0.45	0.27—0.78	0.004	0.62	0.40—0.97	0.038*

Significant results at

***0.1%

**1% and

*5%

The findings further indicate that HIV status was significantly associated with the uptake of VMMC. The uptake of VMMC among participants who knew their status was 2.7 times that among those who did not know their HIV status (APRR 2.75, 95% CI 1.85 – 4.08, *p* < 0.001). In addition, Participants who did not disclose the number of their sexual partners were 38% less likely to take up VMMC compared to those who had one or none (APRR 0.62, 95% CI 0.40—0.97, *p* = 0.038). The prevalence of uptake of VMMC among participants who were aware of VMMC was 7.85 times that among those who were not aware of VMMC (APRR 7.85 95% CI 1.07 – 9.80, *p* = 0.043). Also, participants who were aware that Kira Health Center IV provided VMMV services were 85% more likely to uptake VMMC compared to those who were not aware of the service availability (APRR 1.85, 95% CI 1.85 – 4.08, *p* < 0.001).

## Discussion

The focus of this study was the prevalence and factors associated with VMMC among men of 15–49 years attending the OPD at Kira HC IV in Kira Municipality, Wakiso district. The study findings showed that only about three in every ten men aged 15 – 49 years attending the OPD at Kira HC IV have undergone VMMC. The study findings also show that participants who were married, from tribes in western or eastern Uganda and those that had an undisclosed number of sexual partners, were less likely to have undergone VMMC. On the other hand, men who were aware of VMCC, men who knew that VMMC was free at Kira HC IV and men who knew their HIV status were more likely to have undergone VMMC.

Only three in every ten men aged 15 – 49 years attending the OPD at Kira HC IV have undergone VMMC. The proportion observed by this study is similar to that observed almost 5 years ago by the UDHS at 30.5% [9] but lower than that observed globally at 40% [15]. This may imply that there has not been any remarkable increase in the uptake of VMMC in Uganda and it may be attributed to the fact that circumcision is not a national religious or cultural practice in the country or the region where the study was conducted [2]. The suboptimal uptake of VMMC observed by the study is worrying given that the study area is located in Wakiso, the district with the highest prevalence of HIV in Uganda [12]. This also undermines the investments made by the MoH and its partners in increasing the coverage of VMMC [10, 11] and is an obstacle to the prevention of HIV in this population [18].

The uptake of VMMC was less among married men compared to unmarried men. The observation is similar to that of a qualitative study where they observed a high prevalence of the idea that marriage was protective against HIV [19]. This was due to the notion that married men were less likely to be promiscuous. Similarly, other studies have also shown that the acceptability of VMMC among married men is low [20–22]. Therefore, their VMMC interventions among married men may need to be refined to increase the proportion of married men who undergo VMMC, otherwise, to increase the coverage of VMMC, it may be critical for VMMC interventions to prioritize unmarried men.

The uptake of VMMC was less among participants from Western and Eastern tribes respectively compared to the central region. Circumcision is not practised culturally or religiously in the Western part of Uganda consequently this observation is not alarming [2]. However, in the Eastern part of Uganda, circumcisions are commonly practised culturally and religiously, the majority of circumcisions being non-medical cultural circumcisions [23]. Therefore, the observation made by the study in this regard could be that the majority of persons circumcised in that region are not circumcised under VMMC but rather under routine cultural or religious circumcisions [2]. Similar studies in Zimbabwe [24], Kenya [19] and Botswana [25] also found that cultural practices influenced VMMC practices with the acceptability of VMMC varying by culture. Therefore, although this study observed a lower prevalence of VMMC uptake in both the Eastern Region (where cultural circumcisions are commonly practised) and the Western Region (where there are no widespread cultural circumcisions), the explanation could be two-fold. Men from the Western Region may have low acceptability of VMMC because it’s not a common cultural practice while those from the Eastern Region may have low acceptability due to the preference for cultural circumcisions [23]. Therefore, VMMC programs need to develop cultural-penetrating strategies to increase adoption and acceptability in such a culturally diverse country.

The study findings also showed that the uptake of VMMC was less likely among participants who didn’t disclose the number of their sexual partners than those that had one or no sexual partner at all. This is similar to the observations made from a secondary analysis of data from the Zimbabwe Demographic and Health [26]. An increased perceived risk of contracting HIV due to many sexual partners can increase VMMC uptake by some men [27], the prevention of which is the core message of VMMC programs globally [7] and locally [11]. However, this finding should be interpreted with caution because this study was unable to establish how many sexual partners those that didn’t disclose had.

On the other hand, the study established that VMMC uptake among participants who were aware of VMMC was 7 times that of those who were not aware of VMMC. Knowledge of VMMC is coupled with knowing its advantages in reducing the risk of contracting HIV [11]. Such awareness has been observed to increase VMMC uptake in South Africa [27], Kenya [28] and Zimbabwe [26]. Improving awareness of VMMC is one of the primary strategies that the MoH is using in Uganda to improve VMMC uptake [11]. However, about one in every ten men in this study didn’t have any knowledge about VMMC. Although this was a low percentage thereby commending the efforts of MoH in sharing VMMC information, it is also shown that there is still an information gap that needs to be addressed. On the other hand, the high level of awareness establishes a cause for concern as to why VMMC rates are low (three in every ten men) yet VMMC awareness is high (nine in every ten men). Therefore, this study tries to identify the barriers to VMMC in this regard and bridges the gap in explaining this, albeit to a limited extent. A further qualitative study could examine the reasons why men with VMMC information choose not uptake VMMC in the study setting.

Moreover, this study also found that the prevalence of uptake of VMMC was more among men who knew that VMMC was free at Kira HC IV than those that didn’t know. As discussed earlier, awareness of VMMC is pivotal in increasing VMMC uptake [10, 11, 26–28]. This finding emphasizes the need for men to know where they can find the VMMC services. Although the study findings showed that nine in ten men were aware of VMMC, five in ten men didn’t know that Kira HC IV offered VMMC services. Moving ahead from the MoH communication efforts regarding the communication VMMC service provision at public health facilities [11], the findings suggest a need for health facilities offering VMMC services to cascade the communication to the community.

Finally, the findings of the study suggest that participants who knew their HIV status were 2.7 times more likely to have undergone VMMC as compared to participants who didn’t know their HIV status. This observation is similar to the observations of studies in South Africa [27] and East Africa [29]. Knowledge of HIV status may be linked to the perception of the risk of HIV [19] which may, in turn, increase the odds of VMMC [27]. It is important to note that this may also be complemented by the fact that HIV testing is usually part of the VMMC service provision continuum [11], therefore, it is quite likely that those who have undergone VMMC are likely to know their HIV status. However, on the other hand, seeking VMMC services may be attributed to risk perception [27] which could be linked to a higher ability to seek medical services such as HIV testing given that fear of HIV test results is one of the known barriers against VMMC [27].

Although this study is one of very few to examine VMMC in the context of the OPD within a free VMMC providing public facility, this study had some limitations. First, VMMC uptake and other variables considered for this study were self-reported, therefore, the study findings may be influenced by exaggerated answers and social desirability bias [30,31]. However, the research assistants were trained to probe answers for correctness and to include additional validation checks to confirm the true responses. Also, the study was conducted in only one department, the OPD, at only one health facility, Kira Health Centre IV. Therefore, the findings, also though informative and could help in VMMC programming, do not necessarily reflect the situation at the entire health facility, the district or the country. Therefore, the results should be used with caution and in context. Moreover, the study was a health facility-based cross-sectional study that may not be generalized to the whole population of men in Kira municipality [32]. Also, the independent and dependent variables were simultaneously assessed, and the findings of the study are not able to establish a temporal relationship between the independent and dependent variables.

## Conclusion

There was a low uptake of VMMC among men attending the OPD at the second largest health care facility in Kira Municipality, with only about three in every ten men aged 15 – 49 years attending the OPD clinic at Kira HC IV. The findings showed that among the predisposing factors associated with VMMC uptake, men who were married, from tribes in Western or Eastern Uganda and those that had an undisclosed number of sexual partners, were less likely to have undergone VMMC. Regarding enabling factors for VMMC, men who were aware of VMCC services, men who knew that VMMC was free at Kira HC IV and men who knew their HIV status were more likely to have undergone VMMC. Among the need factors associated with VMMC uptake, the findings showed that participants who didn’t disclose their sexual partner number were less likely to have undergone VMMC.

To increase the uptake of VMMC services at Kira HC IV, the administration of the health centre should collaboratively work with the MoH and VMMC partners to invest in communication approaches to improve VMMC awareness and utilize the OPD as an avenue for providing VMMC education. Since OPD clinics provide an easy catchment population, messages may be developed following the local context at Kira HC IV or any other clinics. To respond to the predisposing factors affecting VMMC uptake in the study population, the VMMC clinic at Kira HC IV, the MoH and other VMMC partners should target men who were married, from tribes in Western or Eastern Uganda and those that had an undisclosed number of sexual partners. Men in these categories were less likely to have undergone VMMC. Interventions could leverage the use of spousal influence, understanding and developing cultural contextual messages towards VMMC adoption.

To strengthen enabling factors for VMMC uptake, the findings of the study highlight a need to intensify VMMC information communication. Men who were aware of VMCC or knew that VMMC was free at Kira HC IV and men who knew their HIV status were more likely to have undergone VMMC. Therefore, the VMMC team at Kira HC IV with support from the administration could ensure a daily delivery of VMMC talks and communication messages at the Kira HC IV OPD. The messages should be coupled with the information that the service is free of charge at the health facility. Among the need factors associated with VMMC uptake, the study findings showed that participants who didn't disclose their sexual partner number were less likely to have undergone it. Therefore, this study recommended the need for VMMC communication teams to continually communicate VMMC benefits regarding the reduction in risk of transmission of HIV.

## Data Availability

The dataset used and/or analysed during the current assessment is available from the corresponding author on reasonable request.

## Abbreviations

AIDS: Acquired immunodeficiency syndrome
APRR: Adjusted Prevalence Risk Ratio
CDC: Centres for Disease Control
COVID-19: Coronavirus Disease of 2019
CI: Confidence Interval
HC: Health Centre
HIV: Human immunodeficiency virus
MOH: Ministry of Health
UBOS: Uganda Bureau of Statistics
VMMC: Voluntary Medical Male Circumcision
WHO: World Health Organization
ODK: Open Data Kit
UPRR: Unadjusted Prevalence Risk Ratio
*P*-Value: Probability Values
SSA: Sub-Saharan Africa

## References

[1] Aggleton P (2007). “Just a Snip”?: a social history of male circumcision. Reprod Health Matters.

[2] Nanteza BM (2020). Enhancers and barriers to uptake of male circumcision services in Northern Uganda: a qualitative study. AIDS Care.

[3] Tobian AAR, Gray RH (2011). The medical benefits of male circumcision. JAMA.

[4] Wamai RG (2011). Male circumcision for HIV prevention: current evidence and implementation in sub-saharan Africa. J Int AIDS Soc.

[5] Gray RH (2007). Male circumcision for HIV prevention in men in Rakai, Uganda: a randomised trial. Lancet Lond Engl.

[6] Bailey RC (2007). Male circumcision for HIV prevention in young men in Kisumu, Kenya: a randomised controlled trial. Lancet.

[7] WHO. World Health Organization | Voluntary medical male circumcision for HIV prevention. *WHO* https://www.who.int/hiv/topics/malecircumcision/fact_sheet/en/ (2012).

[8] Wilcken A, Keil T, Dick B. Traditional male circumcision in eastern and southern Africa: a systematic review of prevalence and complications. Bull World Health Organ. 2010;88(12):907-14. 10.2471/BLT.09.072975. Epub 2010 Oct 29. 10.2471/BLT.09.072975 PMC2995181 21124715

[9] Uganda Bureau of Statistics (UBOS) and ICF. Uganda Demographic and Health Survey 2016. Kampala, Uganda and Rockville, Maryland, USA: UBOS and ICF; 2018.

[10] Ministry of Health Uganda. Safe male circumcision for HIV prevention: national communication strategy. 2010.

[11] Ministry of health Uganda. Safe male circumcision policy; 2010.

[12] Ministry of Health Uganda. Uganda Population-based HIV Impact Assessment (UPHIA) 2016-2017: Final Report. Kampala: Ministry of Health; 2019.

[13] Kira Municipal Council, Uganda. 2021. https://www.kiramc.go.ug/kira-health-centre-iii-elevated-super-health-centre-iv-facility-construction-kicks-meet-standard.

[14] Kish L. Survey Sampling. New York: Wiley; 1965. ISBN 978-0471109495.

[15] Morris BJ (2016). Estimation of country-specific and global prevalence of male circumcision. Popul Health Metr.

[16] Open Data Kit (ODK) - Collect data anywhere. 2020. https://getodk.org.

[17] StataCorp. Stata Statistical Software: Release 15. College Station: StataCorp LLC; 2017.

[18] Davis SM (2018). Progress in voluntary medical male circumcision for HIV prevention supported by the US President’s emergency plan for AIDS Relief through 2017: longitudinal and recent cross-sectional programme data. BMJ Open.

[19] Macintyre K (2014). Attitudes, perceptions and potential uptake of male circumcision among older men in Turkana County, Kenya using qualitative methods. PLoS ONE.

[20] Plotkin M (2013). “Man, what took you so long?” Social and individual factors affecting adult attendance at voluntary medical male circumcision services in Tanzania. Glob Health Sci Pract.

[21] Shacham E, Godlonton S, Thornton RL (2014). Perceptions of male circumcision among married couples in rural Malawi. J Int Assoc Provid AIDS Care JIAPAC.

[22] Osaki H (2015). “If you are not circumcised, I cannot say Yes”: the role of women in promoting the uptake of voluntary medical male circumcision in Tanzania. PLoS ONE.

[23] Sarvestani AS, Bufumbo L, Geiger JD, Sienko KH (2012). Traditional male circumcision in Uganda: a qualitative focus group discussion analysis. PLoS ONE.

[24] Rupfutse M (2014). Factors associated with uptake of voluntary medical male circumcision, Mazowe District, Zimbabwe, 2014. Pan Afr Med J.

[25] Mavundla TR, Mbengo F, Ngomi KB (2020). Perceived influence of value systems on the uptake of voluntary medical male circumcision among men in Kweneng East, Botswana. SAHARA J J Soc Asp HIVAIDS Res Alliance.

[26] Chikutsa A, Ncube AC, Mutsau S (2015). Association between wanting circumcision and risky sexual behaviour in Zimbabwe: evidence from the 2010-11 Zimbabwe demographic and health survey. Reprod Health.

[27] George G (2014). Barriers and facilitators to the uptake of voluntary medical male circumcision (VMMC) among adolescent boys in KwaZulu-Natal, South Africa. Afr J AIDS Res AJAR.

[28] Nyaga EM, Mbugua GG, Muthami L, Gikunju JK (2014). Factors influencing voluntary medical male circumcision among men aged 18–50 years in Kibera division. East Afr Med J.

[29] Lau FK, Jayakumar S, Sgaier SK (2015). Understanding the socio-economic and sexual behavioural correlates of male circumcision across eleven voluntary medical male circumcision priority countries in southeastern Africa. BMC Public Health.

[30] van de Thea F (2012). Faking it: social desirability response bias in self-report research. Aust J Adv Nurs.

[31] Yin RK. Case study research design and methods: applied social research and methods series. Second edn. Sage publications inc.; 1994.

[32] Wang X, Cheng Z, Cross-Sectional Studies (2020). Strengths, Weaknesses, and recommendations. Chest.

[33] Group BMJP (1996). Declaration of Helsinki (1964) BMJ.

[34] von Elm E (2014). The strengthening the reporting of Observational Studies in Epidemiology (STROBE) Statement: guidelines for reporting observational studies. Int J Surg.

